# Russian Financial Statements Database: A firm-level collection of the universe of financial statements

**DOI:** 10.1038/s41597-025-05150-1

**Published:** 2025-06-13

**Authors:** Sergey Bondarkov, Viktor Ledenev, Dmitry Skougarevskiy

**Affiliations:** European University at Saint Petersburg, The Institute for the Rule of Law, Saint Petersburg, 191187 Russia

**Keywords:** Economics, Industry, Business, Geography, Law

## Abstract

The Russian Financial Statements Database (RFSD) is an open, harmonized collection of annual unconsolidated financial statements of the universe of Russian firms in 2011–2023. It is the first open data set with information on every active firm in the country, including non-filing firms. With 56.6 million geolocated firm-year observations gathered from two official sources, the RFSD features multiple end-user quality-of-life improvements such as data imputation, statement articulation, harmonization across data providers and formats, and data enrichment. Extensive internal and external validation shows that most statements articulate well while their aggregates display higher correlation with the regional GDP than the previous gridded GDP data products. We also examine the direction and magnitude of the reporting bias by comparing the universe of firms that are required to file with the actual filers. The RFSD can be used in various economic applications as diverse as calibration of micro-founded models, estimation of markups and productivity, or assessing industry organization and market power.

## Background & Summary

Financial statements are the main source of publicly available information about firms. Any economic analysis based on national or regional aggregates may overlook the underlying heterogeneity of individual firms^1–3^. While many firm- or plant-level surveys have become freely available since the 1990s^4^, including from developing countries^5,6^, country-wide open firm-level datasets are still rare. Instead, scholars resort to commercial databases maintained by business information publishers: Moody’s ORBIS, S&P’s Compustat, or Refinitiv’s Worldscope. However, firm coverage and representativeness of the commercial sources is often low^7^, especially for developing countries and emerging markets^8,9^. Commercial databases are also notorious for the ambiguity in the definition of the unit of analysis, imprecise or missing values, selection and survival bias, reporting lags, lack of transparency, unidentifiable coding errors, steep learning curve, and restrictive access costs^10–13^. These problems can materially affect study results^8,9,14^. Some researchers choose to construct derivative versions of the commercial data sets^15–17^ to overcome these problems while others create their own databases from public or administrative sources^18–21^.

Here we present the Russian Financial Statements Database (RFSD)^22,23^ — an open, harmonized dataset with the universe of annual financial statements filed by Russian firms in 2011–2023. This dataset with 56.6 million firm-year observations is built from administrative data and features a number of end-user quality-of-life improvements such as data imputation, statement articulation, and harmonization across data providers and formats.

Extant literature relying on Russian firm-level data studies corporate governance^24–27^, financial transparency and reporting^28,29^, allocative efficiency^30–33^, firm entry^34^, corruption or embezzlement^35,36^, regulatory capture^37^, access to credit^38^. Firm-level data is also harnessed by researchers in government^39^ and international organizations^24,40^. To date, these and other firm-level studies of the Russian economy have relied almost exclusively on commercial databases: Moody’s Orbis (and its component Ruslana) or Interfax’s SPARK^13^. Problems of data imbalance, under-representation of small and medium-sized enterprises, and missing values loom large in these sources^32,33,41^. For instance, Moody’s Ruslana represents about 10% of total wage employment and poorly covers some industries such as real estate or educational services^13^.

The RFSD responds to the growing demand for open and reliable firm-level data on the Russian economy. Taking careful account of the changes in reporting standards, forms, and rules, we source administrative data on financial statements and unify the information from company balance sheets, income statements, cash flow statements, etc. into a single flat table. We then use the following-year statements to impute missing values in the current-year statements and reconstruct the data wherever possible. We further correct the errors in the financial statements by restating them in accordance with the applicable accounting rules.

Crucially and in contrast to other sources of firm-level information, we enrich the data in the RFSD with the information on non-filers — that is, companies that were legally required to submit their statements and did not benefit from any exemptions but failed to do so. To do this, we gather information on the universe of legal entities registered and active in Russia in 2011–2023 from legally binding administrative data. The information includes primary industry code, address of incorporation, legal form, etc. With this data we define the entities that are mandated to file their financial statements and append them as having missing financials in the RFSD. This way, we are able to understand the magnitude and sign of the reporting bias: are the eligible non-filers systematically different from the filing firms? We observe an alarming pattern: only 44.1% of the expected annual filings by eligible firms in 2011–2023 are present in the administrative data underlying the RFSD. While we are able to reconstruct additional 5.5% of missing filings from next-year statements, failure to file is an important source of selection bias. Firms designated by the government as strategic or firms exiting the market tend not to file. Non-filing is also found to exhibit serial correlation. In contrast, state-owned firms show better filing discipline. To the best of our knowledge, the RFSD is the only openly available country-level dataset with financial statements that includes non-filing firms and thereby allows for explicit handling of non-reporting and selection bias.

## Methods

**Fig. 1:**
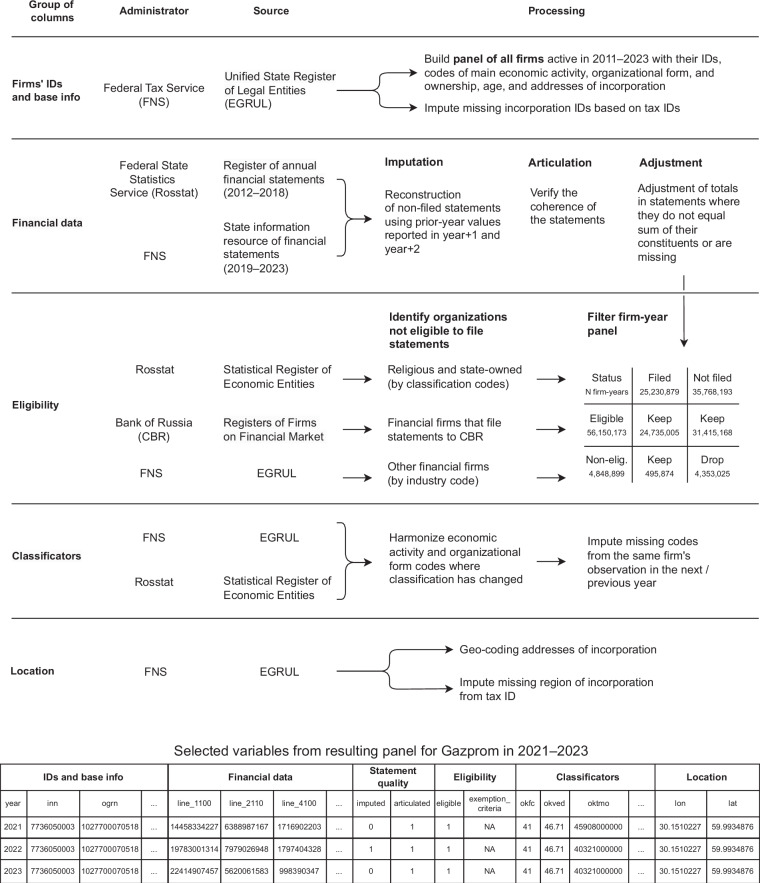
Schematic overview of the RFSD construction.

The process to construct the RFSD is outlined in Fig. 1. Below we provide a step-by-step overview.

### Acquiring information on the universe of firms

The Federal Tax Service of Russia (FNS) administers the Uniform State Register of Legal Entities (EGRUL)^42^. It contains the official and legally binding information on every active organization in the country. The FNS maintains a fee-based Application Programming Interface (API) for this resource (https://www.nalog.gov.ru/rn77/service/egrip2/egrip_vzayim/) — we purchased access to it and gathered the end-of-year snapshots for 2015–2023 that also included organizations that had been dissolved by 2015. Each snapshot is a collection of millions of eXtensible Markup Language (XML) files (one per firm) with basic information such as firm name, taxpayer identifier, address of incorporation, main and secondary NACE Rev. 2-compatible industry codes (OKVED), organizational form (stock corporation, limited liability company, government agency, etc.), date of incorporation or liquidation. We developed parsers for robust extraction of this information from individual XML files and stored it in a flat table with 60,999,072 firm-year observations for the period 2011–2023 where each firm is uniquely identified by a combination of its taxpayer identifier (INN) or organization identifier (OGRN).

### Defining eligible firms

Most Russian organizations are required to file annual unconsolidated financial statements with the Federal State Statistics Service of Russia (Rosstat) before 2019 and with the FNS since 2019. The following entities are exempt from this requirement by law: government bodies and government-owned public service providers such as schools or hospitals, religious organizations, and financial organizations that submit statements to the Central Bank of Russia (CBR), such as banks, insurance companies, brokers^43^. Finally, the firms incorporated in the last quarter of the year are not required to file their statements for that year^44^. The Rosstat maintains the Statistical Register of Economic Entities (https://rosstat.gov.ru/opendata/7708234640-urid1) with the information on government ownership and organizational form for all Russian organizations. The register is publicly accessible (with two caveats: Rosstat’s website is accessible only from Russian IP addresses and also requires users to either use a browser developed in Russia or install security certificates issued by The Ministry of Digital Development and Communications). We extend the information from the EGRUL with these codes and define government and religious entities based on their year-varying organizational form and/or ownership codes. Financial firms are defined following the CBR’s Registers of Professional Participants of Financial Market (https://cbr.ru/registries) supplemented with the list of credit, insurance institutions and investment, private pension funds from the Register of Firms on Financial Market (https://www.cbr.ru/statistics/reporting/lidt_org_fin/). Newly incorporated firms are defined based on their quarter of incorporation. These rule-based exclusions produce 4,848,899 ineligible and 56,150,173 eligible firm-years from the universe of 60,999,072 organization-years active in 2011–2023. Ideally, we would expect all eligible firms to file their annual statements.

### Acquiring financial statements

We collect financial statements from the Rosstat (for 2012–2018) and the FNS (for 2019–2023). The statements filed to the Rosstat are publicly accessible in a tabular Comma-Separated Value (CSV) format (one CSV per year comprising statements from all firms) on its website (https://rosstat.gov.ru/opendata?tag=13). The FNS statements for individual firms are accessible via a fee-based API called GIR BO (https://bo.nalog.ru/). We downloaded the Rosstat’s yearly CSV files and purchased access to the FNS API to query millions of firm-year XMLs with statements for every active firm. It is also noteworthy that we have maintained a record of non-filing firms, that is to say, active firms (in accordance with the EGRUL) that have no financial statements for the corresponding year in the FNS API. Our query was made in late 2024, which was beyond the filing deadline for 2023 (April 1, 2024), and was designed to make multiple requests for firms that the API returned no statements for to minimise data loss. We believe that this way we captured the universe of statements filed by the Russian firms. Then we developed the procedures for robust extraction of information from the two data providers — the Rosstat (CSVs, before 2019) or the FNS (XMLs, from 2019) and formed a flat table with the firm-level financial statements for 2012–2023 where each firm is uniquely identified by its taxpayer identifier (INN). We removed statements for firm-years that had no match in the EGRUL panel of active firms. Our procedures account for the multiple filings per firm-year that occur when firms submit adjusted statements by taking the most recent filing available. Between 160 and 320 thousand firms filed adjusted statements each year in 2019–2023, with some of them revising their statements multiple times.

### Imputation

**Fig. 2:**
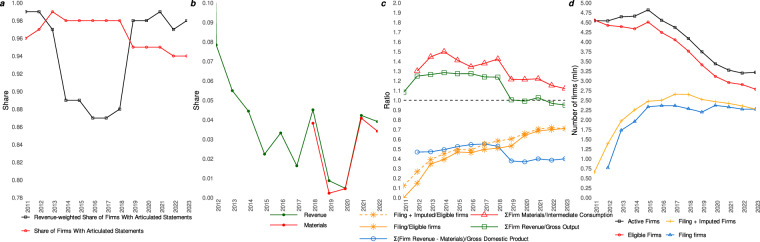
Validation of the RFSD. (**a–d**). Firms with anomalous values are excluded. (**a**) Annual shares and revenue-weighted shares of firms with articulated statements. (**b**) Shares of revenue, materials, and value added imputed from the next-year statements. We define value added as revenue minus materials for firms reporting both non-zero positive variables. (**c**) Filing rate and Gross Output, Intermediate Consumption, or Value Added in the RFSD vs. National Accounts. (**d**) Numbers of active firms in the FNS official bulletin on entity registration vs. eligible or filing firms in the RFSD.

Russian firms are required to state not only the current year financials but also the preceding year financials (in case of the balance sheet variables, two prior year financials) each year. This fact is leveraged to reconstruct missing statements for firms not filing in year *t* but filing in years *t* + 1 or *t* + 2 (for the balance sheet): in such cases, we reconstruct the data from the preceding year values reported in the next-year statement — or a balance-sheet-only observation in cases where the closest statement available is *t* + 2 — and add it to our panel. This imputation was beneficial as it allowed us to reconstruct additional 3,060,732 statements. This approach proved particularly fruitful in the case of 2011, for which no individual statement could be identified within the Rosstat dataset. Figure 2(d) shows that the imputation allows us to restore the data for about 5% firms each year. Figure 2(b), in turn, reports the contribution of the restored revenue and materials to the yearly totals in the RFSD. It is important to note that we only impute the entirely missing statements. If a value_*t*_ in statement_*t*_ differs from value_*t*−1_ reported in statement_*t*+1_ it may be due either to a correction or to a change in reporting standards. As it is impossible to distinguish between the two automatically, we leave the not-missing statements as is and do not use the information from *t* + 1 or *t* + 2 to correct the year-*t* statements. Another thing to note is that a statement reconstruction done in the manner described could not be complete in Rosstat’s years as the CSV files it provides did not feature prior-year values for the entire cash flow statement and some other variables. Finally, financial statements provided by the Rosstat and the FNS differ significantly in the way the missing values are handled. Where the FNS provides XML files that simply lack a field if a firm has not filled it, the Rosstat’s CSV files have zeros in place of missing values. In the latter case it is impossible to distinguish between truly zero values submitted by a firm and absent values. Consequently, for the 2011–2018 period, all zeros were treated as missing data.

### Harmonization

The classification of organizational and legal forms (OKOPF) underwent a change in 2013, while classification of industry codes (OKVED) was modified in 2014. We harmonize the said codes across 2011–2023: in case of OKVED we used the official correspondence table (https://www.economy.gov.ru/material/departments/d18/obshcherossiyskie_klassifikatory_zakreplennye_za_minekonomrazvitiya_rossii/), in case of OKOPF we relied on the order that introduced the changes (https://docs.cntd.ru/document/561235708). In case of some firm-years, classification codes were absent; in these instances, the codes were imputed on the basis of the same firm’s observation in the next or previous year. Furthermore, we harmonized financial statements across the data providers and units of measurement (rubles, thousands of rubles, or millions). We also harmonize the statements across the two report forms available to the firms. Small- and medium-sized enterprises in Russia have an option to file simplified statements with less detailed balance sheet and profit and loss statement and without the cash flow statement^45^. Statement forms remained generally stable throughout the covered period.

### Adjustment of totals

The financial statements comprise a set of variables, which provide a summation (totals) of certain sections. These include non-current assets (line 1100), current assets (line 1200), total assets (line 1600), etc. It is necessary to verify that the values displayed in such totals are equal to the sum of their respective components. To illustrate, line 1100 must be equal to the sum of lines 1110, 1120, and so on up to line 1190. Similarly, line 1600 must be equal to the sum of lines 1100 and 1200. In the event that the discrepancy between the stated total value and the calculated one exceeds 4 thousand rubles (the threshold for this discrepancy is derived from the FNS recommendations for statement articulation verification^46^), or is absent, the calculated value is substituted in its place. Additionally, we add the calculated total lines that are not included in the simplified statements form to ensure their compatibility with the full statements.

### Data scope

We have the universe of 60,999,072 organization-years active in 2011–2023 from the EGRUL, out of which we defined 56,150,173 eligible firm-years. We collected 25,230,879 firm-year financial statement filings from the Rosstat or the FNS for the corresponding period and matched them with the universe of firms on the taxpayer identifier (INN) and year. The lion’s share, i.e. 24,735,005 of firm-year filings, comes from the eligible firms. A further 495,874 filings are contributed by the firms we defined as non-eligible to file the statements. This is due to errors on the part of the firms (e.g. small government or religious agencies mistakenly filing their statements), errors in classification codes (when an organization is erroneously defined as government based on its organization code), or changes in the exemption requirements (e.g. some financial firms reporting to the Rosstat instead of the Central Bank in certain years). Importantly, we also have the information on eligible non-filers, or 31,415,168 firm-years that we deemed as eligible but who failed to submit their statements. Finally, as expected, we detect 4,353,025 non-eligible firm-years that have no filings. We exclude such non-eligible non-filers from the data set altogether. These are mostly government agencies, religious, or financial entities who are not required to file their accounts to the Rosstat or the FNS. We arrive at a panel with the universe of the eligible firms (regardless of their filing status) as well as the non-eligible filers in 2011–2023 with a total of 56,646,047 firm-year observations for 9,560,262 firms.

### Data enrichment

We conducted location inference using the addresses of incorporation reported in the EGRUL for every firm-year since 2014 (end-year of our last EGRUL snapshot). The fact that the addresses are stored in a structured form, with separate fields for region, city (or village), street, and house names or numbers, provides a significant advantage. We set up a local instance with OpenStreetMap Nominatim v. 4.4.0^47^, a fast, scalable, and open geocoding solution, using a Docker container provided by https://github.com/mediagis/nominatim-docker. Then we performed structured queries to Nominatim to geocode every unique address of incorporation in the EGRUL. The initial query included all the constituent elements of the address, including the region, city, street, and house number. In the event of unsuccessful geocoding, the house was subsequently excluded from the second query. In the event of a failure, a third query was conducted, utilising solely the region and city names. Subsequently, the obtained geographic coordinates were divided into three categories based on their Nominatim Address Rank. The Address Rank is a value that ranges from 4 to 30 and can be converted back into a specific component of a structured address (for example, 4 represents a country and 30 represents a house). Addresses with a rank of 30 were considered to have been geocoded up to the level of the house, while addresses with a rank between 26 and 29 were treated as having been geocoded up to the level of the street. Addresses with a rank between 12 and 25 were regarded as having been geocoded at the city level.

### Variables

Consider an extract from the RFSD at the bottom of Fig. 1 for Russia’s largest company, Gazprom, in 2021–2023. Each company in the data is identified by either the taxpayer identifier (INN) or the organization identifier (OGRN), with the former being the preferred option due to its use in the FNS API. The data then includes 187 variables from the financial statements: balance sheet (line_1100–line_1700), profit & loss statement (line_2100–line_2910), statement of changes in equity (line_3100–line_3600), cash flow statement (line_4100–line_4490), and statement on the proper use of funds received (line_6100–line_6400). The numbers in variable names correspond to the official codes of the variables^48^ such that line_1100 are non-current assets, line_2110 is revenue, and line_4100 is cash flow from operating activities. The full table with English-language names and descriptions of the variables is available in Table A.1 in the Supplementary Materials. Apart from these regular lines, there are optional (decoding) lines which a firm may use to further detail its statement. Unlike the regular variables, these have no dedicated numbers and are named as the firm sees fit. To illustrate, Gazprom’s profit and loss statements detail its revenue in line_2110 by source in the decoding lines: revenue from gas sales, oil sales, petrochemicals sales. Since both the naming and the logic behind decoding is not uniform, it is not feasible to include these optional lines in a flat table (and because of that this information is absent in the Rosstat’s CSVs, 2012–2018, and only present in the FNS XMLs, 2019–2023). However, cash flow statements do not articulate if such lines are present and one does not take them into account. This poses a problem since the decoding lines are widely used by larger firms, present in more than 200,000 observations in 2019–2023 with average revenue close to 2 bln rubles. We parse the optional lines present in cash flow statements — to name them we use an x suffix in place of a last digit that a line’s name would have if it was a regular line, e.g. line_411x (in cases where several additional variables were used to detail the same item, we provide the sum of them). We do not do the same for the balance sheet and other parts of a statement (with the exception of changes in equity), since the structure of these are such that optional lines would simply sum up to the value in an item they decode, and inclusion of the sum of them is therefore redundant. We also flag the statements that were missing but that we were able to impute from the next-year filings. Gazprom, in particular, did not file in 2022, and the statements for that year are reconstructed from the 2023 filings. Apart from the identifiers, the basic information from the EGRUL or other registers is added, such as the primary industry or organizational form codes, or firm age. Finally, we have the longitude and latitude of the firm address of incorporation and its level of detail.

## Data Records

The Russian Financial Statements Database^22^ is available at Zenodo^22^ and Hugging Face^23^. The data is stored in a structured, column-oriented, compressed binary format Apache Parquet^49^. It is partitioned by year enabling end-users to query only variables of interest in years of interest without loading the full data into memory. The current version of the RFSD is 1.0.1 (SemVer). We intend to update the database annually as the new data becomes available, in other words when most of the firms have their statements filed with the Federal Tax Service.

## Technical Validation

We conduct an internal and external validation of the RFSD. Internally, we check whether the financial variables are logically consistent and sum to the known amounts. Externally, we check whether the aggregates from the RFSD align with the aggregates from independent official or academic sources.

### Internal validation

#### Articulation

Financial statement articulation is the relationship between its constituent parts that is both logically and mathematically consistent. We perform internal validation of the RFSD by checking whether the individual statements articulate well. In particular, we equate the total values in the summarizing lines (e.g. non-current assets (line 1100), total assets (line 1600), etc.) with the manually calculated totals of their constituent parts. The manual calculation is made following the official guidelines for financial statement articulation by the FNS^46^. These guidelines define 67 equations for firms submitting full statements and 38 equations for firms filing simplified statements pertaining to different parts of the statement. We use only equations for the balance sheet, profit and loss, and cash flow statements parts — 22 equations for full statements and 4 for simplified statements. The full list of equations is available in Table A.2 in the Supplementary Materials. We flag a financial statement as articulated if the discrepancy for every applicable equation is within the official threshold of 4 thousand rubles (about USD40 as of late 2024) defined by the FNS^50^. We find that only about 5% of statements do not articulate (see Fig. 2(a) for a temporal evolution). We observe that the share of articulating statements was high in the Rosstat data (2011–2018) and decreased slightly following the change in the data provider to FNS (2019–2023). These trends might be misleading, however, because lack of articulation by a large firm might matter more than the erroneous statement filed by a small firm. In light of this, we additionally report revenue-weighted share of articulating statements each year in Fig. 2(a). Revenue-weighted articulation plummeted in 2014 and rapidly increased in the FNS period starting from 2019. We attribute the 2014 drop to changes in accounting rules that tightened the eligibility criteria to submit simplified statements^51^. This change forced firms to switch to much more detailed full statements that included the decoding lines explained above. The Rosstat data does not handle the decoding lines correctly for many firms, including major companies. The 2019 increase follows the change of the data provider to the FNS and reflects better treatment of the decoding lines in the source data.

### Anomalous values

Internally valid financial statements not only display consistent relationships between their constituent parts, but also report reasonable values. This is especially relevant for the RFSD where we observed two firms reporting revenue that was larger than Gazprom or Rosneft, Russia’s largest companies by revenue and capital, by a factor of 8 to 26. Since having such anomalous values is detrimental to any aggregation, we engaged in manual review of top-20 firms in terms of revenue or total assets within each 2-digit industry (excluding financial firms), firms with largest year-on-year changes in key financials, and firms with imputed statements and largest revenues. Our review has identified 436 firms that filed 1,130 anomalous statements in 2011–2023. The judgement was made based on the audit opinions, financials of known industry leaders, firm websites, or public information regarding the firms suspected of reporting abnormal values. We recommend the RFSD users to exclude those companies from consideration and do so in our external validation.

Our manual review has produced an expert-made list of anomalous filing. Instead, one might consider classical or machine learning-based algorithms for outlier detection to increase the objectivity of the results. Hawkins^52^ defines an outlier as an observation that deviates so significantly from the rest of the data that it raises suspicion it was produced by a different data generating process. We applied five well-established classical outlier detection algorithms to the RFSD data, considering each second-digit industry and year as a separate data set. Such an industry-year stratification was necessary to account for high between-industry variation in financials (e.g., petrochemical producers will report much larger financials than furniture manufacturers), inflation, and temporal improvements in filing quality. As in our manual review, we considered Hawkins outliers only in terms of revenue and total assets, searching for anomalies in 19,377,043 firm-year observations of 3,844,541 firms reporting both variables in 2011–2023. We then compared the manual outlier classification with the outlierness scores of five statistical algorithms by calculating the Receiver Operating Characteristic Area Under the Curve (ROC AUC) for each year and averaging it over the period. As most algorithms require the neighbourhood size *K* to be specified, we used the Natural Neighbour algorithm^53^ to determine the optimal *K* for each industry-year before applying the outlier detection algorithms. All calculations were performed using the DDOutlier package^54^ in R^55^. The results are in Table 1. We immediately see that most of the algorithms are in relatively high agreement with manual classification, judging by the AUC in the 0.8–0.9 range, with HiOut^56^ reporting the highest conformance. However, the key challenge in relying on statistical outlier detection is that one still needs to specify a threshold of outlierness score to treat an observation as an outlier. This problem is highlighted in columns 2–8 of Table 1. If we set the score threshold at the minimum value of the score among the manually identified outliers, each algorithm will classify 1.2–1.4 million firms as outliers each year, which is unrealistic. If the equate the threshold to the median score among the manually identified outliers, we will classify 1.5–3.6 thousand firms as outliers by any algorithm, except for KDEOS^57^. These results emphasize that while the statistical outlier detection algorithms are producing objective results, threshold selection is still an expert-driven process requiring supervised data. Furthermore, the experts may better identify Hawkins outliers since they rely on external data such as audit opinions in their manual review. For these reasons we elect to rely on manually defined outliers in our coding.

**Table 1:** Comparison of expert-made RFSD outlier detection with classical algorithms in 2011–2023.

Algorithm	ROC AUC	# Firms with outlierness score exceeding the set threshold from manual anomaly detection					
		Min	25th percentile	Median	Mean	75th percentile	Max
LOF^87^	0.857 ± 0.033	1,396,696 ± 243,033	205,268 ± 179,918	2,033 ± 1,690	244 ± 95	293 ± 95	35 ± 16
HiOut^56^	**0.908 ± 0.019**	1,295,290 ± 264,755	70,708 ± 49,004	1,468 ± 681	240 ± 84	248 ± 117	12 ± 5
LDF^88^	0.855 ± 0.034	1,411,779 ± 219,627	202,914 ± 182,567	1,802 ± 1,325	9,221 ± 5,041	283 ± 103	44 ± 20
RKOF^89^	0.857 ± 0.032	1,407,689 ± 234,609	207,209 ± 161,659	3,635 ± 4,687	29 ± 19	271 ± 96	26 ± 16
KDEOS^57^	0.803 ± 0.022	1,473,802 ± 202,263	326,845 ± 116,560	104,786 ± 37,011	269,959 ± 46,568	80,399 ± 33,299	71,190 ± 32,181

Column “ROC AUC” reports the yearly average AUC for the algorithm-derived outlierness scores vs. the expert-made binary anomaly classification with 95% CIs. Columns “Min”–“Max” report the yearly average number of firms treated as outliers based on their outlierness scores with 95% CIs. In column “Min” this threshold is equal to the minimum value of the outlierness score for the expert-classified anomalous filings, in column “25th percentile” — to the 25th percentile of the outlierness score for expert-made anomalies, etc.

### External validation

#### Comparison with official aggregates

To validate the RFSD externally, we leverage Russia’s National Accounts. As our numerator we take the annual sum of revenue, materials, or value added (defined as revenue minus materials) for all non-anomalous firms reporting non-zero positive values in the RFDS. As the denominator we use the Gross Output, Intermediate Consumption, and Gross Domestic Product, respectively, from the National Accounts^58^. If the resulting ratio is close to one this means that firm-level data aggregates well to the National Accounts. We acknowledge that this comparison is inherently flawed as the two sources are fundamentally different: the unconsolidated financial statements are reported according to the Russian accounting rules on book value while the National Accounts are compiled based on the System of National Accounts rules and are valued at market prices. Gross Domestic Product also accounts for shadow economy and non-market production that may be missing in the RFSD. Figure 2(c) reports the resulting ratios. We find that the RFSD aggregates follow the National Accounts of the Russian economy, with Gross Output and Intermediate Consumption ratios exceeding unity in the Rosstat data before 2019 and closer to unity in later periods. This decrease can be explained by non-filing by country’s largest firms and by sanctions-related legislation allowing certain companies to not publish their statements^59^. This is evidenced if we look at the filing ratio, that is the ratio of filing to eligible firms, in Fig. 2(c). It displays steady increase throughout the years. GDP ratio, in contrast, shows that the value added in the RFSD comprises only 40%-50% of the GDP in the National Accounts. This should not be viewed as indication of data deficiencies and is explained by the aforementioned differences in reporting in the System of National Accounts.

Apart from the National Accounts, we validate the RFSD against the FNS statistics. In Fig. 2(d) we compare the annual count of active firms from the FNS official bulletin^60^ with the number of eligible and filing firms in the RFSD. Each year 280,667 firms are deemed as ineligible on average, and this relationship remains stable over time starting from 2013. We attribute small differences between the counts in 2011–2012 to erroneous extraneous observations in our EGRUL data. The number of filing firms increases with time, suggesting better compliance with the filing requirement in later years. Conversely, the number of active or eligible firms displays a steady decrease since 2016. This is due to the effort of the FNS to identify and liquidate inactive or fly-by-night firms established for tax evasion and managerial diversion purposes. Prior to 2016 approximately 32% of all firms were reportedly identified as rogue, while by the end of 2019 their share had drastically decreased to 3.1%^61^. Finally, Fig. 2(d) shows the beneficial effect of our imputation procedure as 235,441 firms have their statements restored from the next-year filings on average each year.

#### Comparison with Orbis

Moody’s Orbis is the primary source of firm-level information for developed and developing countries^10,16,62^. Its component for Russian, Ukrainian, and Kazakhstan companies, called Ruslana, is sourced, *inter alia*, from the same administrative data provided by the Rosstat and the FNS that we use to construct the RFSD. It is therefore of value to compare data completeness of the RFSD with that of Orbis, especially in light of its ubiquity in the literature. We queried Orbis to extract all Russian entities with known global ultimate owner and over $1 million in revenue in 2021, excluding public authorities. Our query was made in April, 2023 and included only active companies with known financial data for 2021^63^. We impose these restrictions on the Orbis sample due to infeasibility of exporting the full list of all Russian organizations. We then match the Orbis sample with the RFSD on taxpayer and organization identifiers and compare the coverage for 2021 in Table 2. Out of 185,222 firms with financials for 2021 available in our Orbis sample, the vast majority of firms (182,641, 98.6%) also have their financials in the RFSD for 2021. The firms present in both data sets had over $2 trillion of total revenue or assets, forming the bulk of the Russian economy in 2021. The RFSD also includes 58,835 firms reporting over $1 million in revenue in 2021 that are missing in Orbis (second row of Table 2). These missing firms have mean and median revenue and assets comparable to the present firms. Large number of missing firms in Orbis *vis-à-vis* the RFSD highlights that the latter source has non-trivial amount of additional data not present in Orbis. Finally, 2,581 firms with financials in Orbis did not have financials in the RFSD and were flagged as non-filers. These firms were responsible for a non-trivial amount of total revenue ($209 billion) and had larger revenue and total assets on average, suggesting non-random data omissions in the RFSD. We manually examined the leading missing firms in terms of revenue and found that their financials were retrospectively excluded from the FNS API. Starting from financial statements for 2018, major Russian firms were authorized not to disclose their financial statements^59^. The list initially included 11 firms^64^, but after 2022 was expanded to cover more than 1,000 firms^65,66^. We believe that the observed retroactive redaction of the FNS API data and missing financials in the RFSD are explained by firms exercising their right not to disclose.

**Table 2:** Comparison of the RFSD with Orbis in 2021.

Has financials		N Firms	Revenue			Total assets		
RFSD	Orbis		Sum, bln $	Mean, thou $	Median, thou $	Sum, bln $	Mean, thou $	Median, thou $
Yes	Yes	182,641	2,144	11,742	2,212	2,077	11,375	1,153
Yes	No	58,835	802	13,643	2,328	855	14,533	1,345
No	Yes	2,581	209	81,091	7,682	290	112,439	6,969

We consider Russian non-government firms with over $1 million in revenue in 2021 in the two data sets and report the total, mean, and median value of revenue and assets for firms based on their presence in the data sets. Firms with anomalous values are excluded.

#### Spatial comparisons

**Fig. 3:**
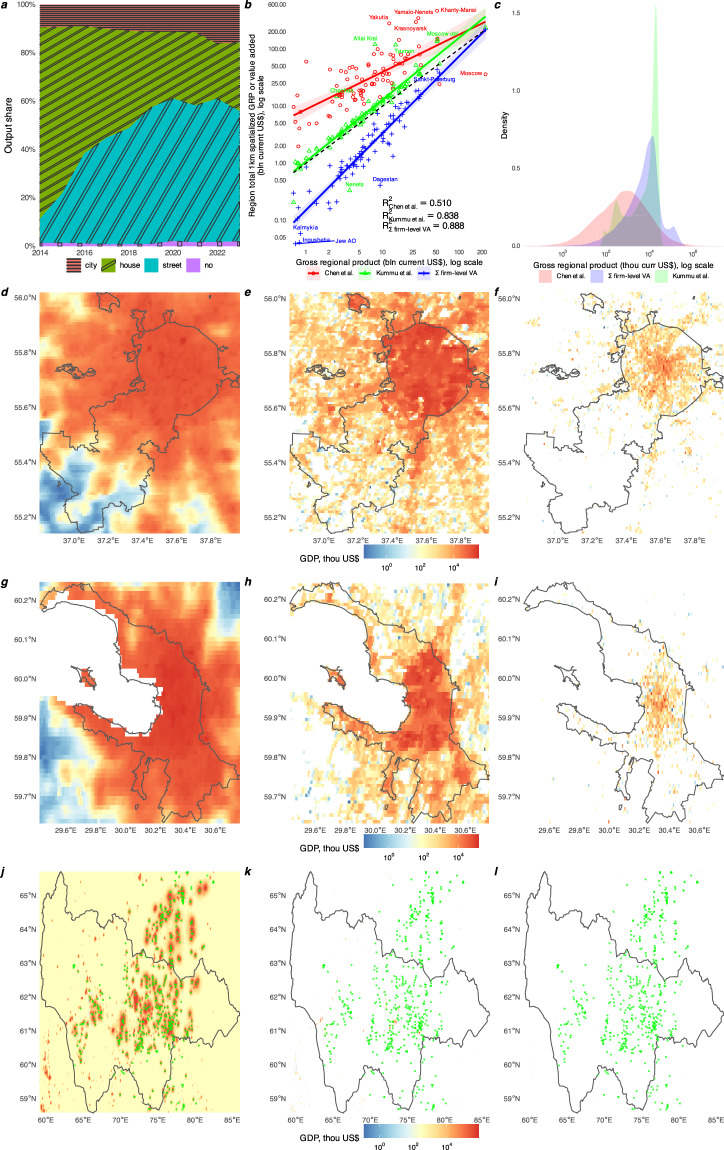
Spatial validation. (**a–l**). (**a**) Revenue-weighted share of Russian firms in 2011–2023 by geocoding level of their address of incorporation. (**b**) Official gross regional product in 2015 and regional totals of its 1 km × 1 km spatializations in 2015 from Chen *et al*.^68^, Kummu *et al*.^67^, or firm-level value added totals for geocoded firms from the RFSD. Coloured solid lines are trends from linear log-log regressions, shaded areas are 95% CI, R^2^ from these regression are reported. Black dashed line is ideal alignment. Regions displaying largest absolute difference with the official data are annotated. (**c**) Kernel density estimates of pixel-level 1 km × 1 km GDP in 2015’s Russia from Chen *et al*., Kummu *et al*., or RFSD firm-level value added on Kummu *et al*. grid. Non-zero pixels only. (**d**) Chen *et al*. non-zero data, 1 km × 1 km, Moscow, 2015. (**e**) Kummu *et al*. non-zero data, 1 km × 1 km, Moscow, 2015. (**f**) RFSD geolocated firm-level non-zero value added, 1 km × 1 km, Moscow, 2015. (**g**) Chen *et al*., Saint Petersburg. (**h**) Kummu, Saint Petersburg. (**i**) RFSD, Saint Petersburg. (**j**) Chen *et al*., Khanty-Mansia. Green dots are gas flare locations in 2015 from World Bank Global Gas Flaring Tracker.^69^ (**k**) Kummu *et al*., Khanty-Mansia. (**l**) RFSD, Khanty-Mansia. Regional boundaries are due to geoBoundaries^90^.

Financial statements in the RFSD are enriched with locational information regarding the address of incorporation. Figure 3(a) reports the revenue-weighted share of firms by geolocation quality. We find that throughout 2014–2023 88.8% of total revenue is geocoded up to a house or street on average in the RFSD; location of 10.0% of revenue is available at city level only, the remaining 1.2% of revenue is impossible to geolocate. We then proceed to assess the validity of the geocoding by comparing the spatially aggregated value added (defined as revenue minus materials) in the RFSD with widely used sources of 1 km × 1 km gridded GDP data from Kummu *et al*.^67^ or Chen *et al*.^68^ for Russia in 2015. In Fig. 3(b) we report region-level aggregates from the three spatializations on the *y*-axis versus the Rosstat’s official reported Gross Regional Product for 2015 on the *x*-axis. It is immediately evident that Chen *et al*. data product is ill-aligned with the official data in Russia. For instance, in 2015 Chen *et al*. reported mere $37 billion GDP for Moscow in stark contrast to the official Gross Regional Product (GRP) of $223 billion (all values henceforth are converted to 2015 nominal USD). Error in the opposite direction is observed for the oil-producing region of Khanty-Mansia, where Chen *et al*. report GRP of $474 billion, while the official GRP is only $52 billion. Extreme upward bias of Chen *et al*. is further confirmed when we compare regional aggregates with Kummu *et al*. data product. Regression of log aggregate spatialized GRP on log official GRP reveals that the RFSD spatialization has the highest share of variance explained, with Kummu *et al*. being a close second, and Chen *et al*. a distant third. Two things contribute to the large upward bias of Chen *et al*. spatialization and slight inferiority of Kummy *et al*. data product in relation to the RFSD. First, it is the mechanical imputation of GDP in uninhabited areas by Chen *et al*. Consider the raw 1 km × 1 km pixels for Moscow or Saint Petersburg in Fig. 3(d–i) for Chen *et al*., Kumu *et al*., and the RFSD spatializations, respectively, for 2015. Chen *et al*. spatialization reports non-zero economic activity in almost every land pixel, while Kummu *et al*. and RFSD feature much more pixels with zero GDP. Additionally, the RFSD produces much more focused locations with non-zero value added as suggested by Fig. 3(c) with density of non-zero pixels in the three data sources in Russia in 2015. This should come as no surprise since the RFSD relies on addresses of incorporation which are located in the settled areas. Second, inadequate handling of gas flares — combustion systems utilized in oil wells to incinerate flammable gases, predominantly methane, that are released during the oil extraction process, — by Chen *et al*. also contributes to the upward bias of their data product. Consider the raw pixels for Khanty-Mansia from the three data sources in Fig. 3(j–l), with gas flaring locations identified by the World Bank^69^ for 2015 superimposed as dots. Most economic activity in Chen *et al*. spatialization in Khanty-Mansia is misrepresented as being situated in the sites where gas flaring are also observed. Given that Chen *et al*. data is ultimately based on nighttime lights, we view lack of gas flare filtering as a serious drawback of this resource. In contrast, both Kummu *et al*. data product and the RFSD are free of the gas flaring bias.

### Reporting bias

We observe gradual increase in the proportion of eligible firms filing their financial statements between 2012 and 2023 in Fig. 2(c). However, the average filing rate is still only 71.2% in 2023, meaning that almost one third of eligible firms neglected their duty to file. The extant literature does not reach a consensus on whether private companies tend to report statements of lower quality,^70–72^ although corporate governance studies of Russian firms support the notion that small^29^ and private companies^28,73^ tend to under-report their financials. Non-filers may comprise abandoned or fly-by-night firms contributing to the shadow economy. In addition, we observe regional disparities in reporting, with Ingushetia, Chechnya, and Dagestan demonstrating a substantially lower filing rate (49.6%, 48.7%, 43% respectively) than the national average in 2023. Firms in the capital city also are less likely to file: only 62.2% firms in Moscow filed in 2023.

#### Covariates of reporting

We estimate linear probability models, regressing filing by eligible companies, statement articulation if filed, and reporting anomalous values if filed on selected firm characteristics in 2012–2023. *Strategic*_*i*,*t*_ indicates whether an *i*-th firm is on the list of strategic companies^74,75^ authorized not to disclose their financial statements for year *t*. By the end of 2023 the list of strategic companies included 1,133 firms. *Sanctioned*_*i*,*t*_ indicates if a firm in under sanctions imposed by the international community. This variable is constructed by matching the time-varying lists of sanctioned entities from OpenSanctions, an international database of persons and companies of political, criminal, or economic interest,^76^ with the RFSD on taxpayer or organization identifiers or firm names. In case of matching on firm names we performed a manual match to ensure that we flag the correct entities as being under sanctions. We consider only the sanctions ever imposed on 3,643 firms by the Group of Seven Countries, Australia, New Zealand, and Switzerland as the most consequential ones. Both sanctioned and strategic companies are authorised not to disclose their financial statements^66^, but the two characteristics do not overlap: 55.7% of firms designated as strategic were not under sanctions. To better understand the interaction between the two, we include an additional *Strategic*_*i*,*t*_  ×  *Sanctioned*_*i*,*t*_ term to flag strategic firms that are also under sanctions. *Exit*_*i*,*t*_ indicates whether a firm is liquidated on any ground, that is, it exits a market in year *t*. We include this variable to capture lack of incentives to report after firm exit. *State*-*owned*_*i*,*t*_ indicates whether a firm is directly or partially owned by the state, judging by its classifications codes from the EGRUL or the Statistical Register of Economic Entities. The first and the second models also include lagged dependent variables to explore possible serial correlation in decision-making. In consideration of the presence of lagged variables we exclude newly-incorporated firms in their first year of activity from estimation sample in all models. We are also mindful of the possible multicollinearity between *Strategic*_*i*,*t*_ and *Sanctioned*_*i*,*t*_, given large overlap of the two lists. For this reason we include those two characteristics in stepwise fashion in regression models.

Table 3 presents the results. Filing is negatively associated with being a strategic firm. Statistically and economically significant negative coefficient for filing by strategic firms of −0.230 (p-value: < 0.001) in model (3) confirms systematic under-representation of information by the largest Russian companies in the RFSD that we initially uncovered during comparisons with Orbis. Given that mean filing rate in the sample is 49.4%, we observe a 53.4% decrease of filing for stategic firms. Being under sanctions, however, is not directly associated with non-filing in our saturated model (3) in Table 3 (p-value: 0.068). Instead, being sanctioned offers a weak mediating effect for strategic firms (p-value: 0.016). This indicates that small- and medium-sized firms under sanctions but not deemed strategic still have the incentives to file. The absence of incentives to disclose financial information for liquidated firms is our leading explanation for the observed failure to file among the exiting firms, with statistically significant coefficient of -0.313 in model (3) (p-value: < 0.001). In contrast, state-owned eligible firms demonstrate a statistically significant higher level of discipline and are more likely to file (coefficient: 0.040 in model (3), p-value: < 0.001). Filing also exhibits strong serial correlation (coefficient: 0.602 in model (3), p-value: < 0.001).

**Table 3:** Reporting bias.

Model	(1)	(2)	(3)	(4)	(5)	(6)	(7)	(8)	(9)
	Filed_*i*,*t*_			Articulated_*i*,*t*_			Anomalous_*i*,*t*_		
Filed_*i*,*t*−1_	0.6016^***^	0.6017^***^	0.6016^***^						
	(0.0054)	(0.0054)	(0.0054)						
Articulated_*i*,*t*−1_				0.5888^***^	0.5888^***^	0.5888^***^			
				(0.0170)	(0.0170)	(0.0170)			
Strategic_*i*,*t*_	−0.2647^***^		−0.2304^***^	0.0278***		0.0279***	−7.28 × 10^−5^^***^		−7.26 × 10^−5^^***^
	(0.0154)		(0.0193)	(0.0029)		(0.0027)	(1.97 × 10^−5^)		(2.04 × 10^−5^)
Sanctioned_*i*,*t*_		−0.0488^**^	−0.0224^.^		−0.0087	−0.0088		−8.25 × 10^−5^^*^	−8.25 × 10^−5^^*^
		(0.0162)	(0.0121)		(0.0084)	(0.0084)		(3.42 × 10^−5^)	(3.43 × 10^−5^)
Strategic_*i*,*t*_ × Sanctioned_*i*,*t*_			−0.0576^*^			0.0073			7.63 × 10^−5^
			(0.0233)			(0.0107)			(5.71 × 10^−5^)
Exit_*i*,*t*_	−0.3127^***^	−0.3127^***^	−0.3127^***^	0.0192^***^	0.0192^***^	0.0192^***^	−1.61 × 10^−5^	−1.61 × 10^−5^	−1.61 × 10^−5^
	(0.0240)	(0.0240)	(0.0240)	(0.0013)	(0.0013)	(0.0013)	(1.37 × 10^−5^)	(1.37 × 10^−5^)	(1.37 × 10^−5^)
State-owned_*i*,*t*_	0.0395^***^	0.0390^***^	0.0395^***^	−0.0062	−0.0062	−0.0062	9.91 × 10^−7^	1.02 × 10^−6^	1.06 × 10^−6^
	(0.0060)	(0.0061)	(0.0060)	(0.0039)	(0.0039)	(0.0039)	(2.58 × 10^−5^)	(2.58 × 10^−5^)	(2.58 × 10^−5^)
Sample	ELIGIBLE FIRMS AGED > 1			FILING FIRMS AGED > 1			FILING FIRMS AGED > 1		
Dep. var. mean	0.494			0.962			3.61 × 10^−5^		
N (firm-years)	43,048,385			22,727,193			22,727,193		
Year FE	Yes	Yes	Yes	Yes	Yes	Yes	Yes	Yes	Yes
Industry FE	Yes	Yes	Yes	Yes	Yes	Yes	Yes	Yes	Yes
Region FE	Yes	Yes	Yes	Yes	Yes	Yes	Yes	Yes	Yes
R^2^	0.501	0.501	0.501	0.373	0.373	0.373	0.000	0.000	0.000

Coefficients from ordinary least squares regressions of statement filing, articulation, or anomalous values in the RFSD for 2012–2023 on firm characteristics, excluding firms in their first year. All specifications include year, 2-digit primary industry code, and region of incorporation fixed effects. Huber-Eicker-White standard errors clustered at the region of incorporation are in parentheses. Stars show significance: *p* < 0.001 ***, *p* < 0.01 **, *p* < 0.05 *.

Apart from filing, we study articulation of filed statements. In model (6) in Table 3 we uncover that not sanctioned strategic firms tend to file the statements slighlty more articulate (p-value: <0.001). Finally, we consider the filing of anomalous or implausible values for 436 firms we identified. Submitting anomalous statements is found to have little to no association with firm characteristics (apart from being strategic). This supports the notion that anomalous values are due to random errors.

## Usage Notes

The RFSD is built from official administrative data, which may have inherent flaws and limitations^15^. As described in the Methods section, we address potential issues with financial data by reconstructing the non-filed statements, correcting errors in the reported summation lines, clearly marking anomalous values, and providing both internal and external validation for the resulting data. The official classification codes we rely on in this dataset may also contain missing values and errors. Unfortunately, it is not feasible to validate the official codes, as all the official aggregates we could use as a reference rely on the same official registers. However, there are reasons to believe that the missing values we observe do not introduce significant bias into the RFSD.

Most of the classification codes in this dataset are self-reported by firm at the time of its official registration, in accordance with the submitted documents. This process is partly automated, as in the case of the territory code (OKTMO), and there is little room for error when choosing between legal form (OKOPF) or ownership structure (OKFS) types. An important exception is a firm’s main industry code (OKVED), which is chosen by the firm and may become irrelevant over time if the firm changes its operations but fails to update its industry code. It can also be poorly chosen initially, as OKVED is more complex than other classifiers and is characterized by multiple correspondences between codes and types of economic activities. To the best of our knowledge, there is no publicly available assessment of the scale of miscorrespondence between firms’ main OKVED and their actual main activities. However, there are monetary incentives for firms to keep their OKVED codes up to date. First, each year, a firm is obliged to confirm its main economic activity type to the Social Insurance Fund of the Russian Federation, after which the Fund assigns the corresponding class of professional risk — this, in turn, defines the firm’s insurance premium rate for mandatory social insurance against industrial accidents and occupational diseases. The procedure^77^ requires a firm to provide the proof that the majority of its revenue comes from its stated main industry code. If the firm does not comply, its insurance premium rate is set to the maximum for its assumed activity class. Second, the correct OKVED code may be crucial for obtaining government support, as was the case during the COVID-19 pandemic in Russia, when many firms did not receive support precisely because of ineligible industry codes^78^.

Regarding the missing classification codes, they are highly prevalent in the 2011–2013 period (16-37% of firm-years with missing data), but their share diminishes rapidly for all the classifiers afterward, mostly remaining under 1% in the later part of the period (OKPO identifier is the only exception; however, for most use cases, one would prefer to use OGRN or INN, which are also unique firm identifiers and have no or almost no missing values). To better assess the problem, we calculated the share of total yearly revenue from firms with missing codes, the results leads us to believe that most of the missing values are due to abandoned or fly-by-night firms that were subsequently removed from the EGRUL. For instance, in 2011, where 24% of firms lacked an OKVED code, their combined share of total revenue amounted to just 1%. This figure halved by 2014 and later remained mostly under 0.1%. In conclusion, apart from research focusing on very specific types of economic activity that have multiple correspondences in OKVED, we find the RFSD to be an appropriate source for applications requiring groupings by classification codes.

There are several limitations that one must be aware of when working with RFSD. First limitation is due to the failure to report financials. According to the extant literature and our analysis, small and private companies tend to under-report^28,29,73^. We also find that strategic and sanctioned firms are less inclined to make their financial statements public. Strategic and sanctioned companies tend to be much larger than other firms^79,80^. Second limitation is that the RFSD’s unit of analysis is a legal entity filing an unconsolidated financial statement. It means that intra-group transactions can be doubly-counted in financial statements of legally separated but economically unified units. Third, the RFSD includes only primary industry codes (OKVED). It might lead to aggregation bias due to multi-product firms or incorrect self-assignment of OKVED codes by firms. Fourth, Russian accounting rules remained generally stable throughout the period covered by the RFSD. However, in 2019 the full form of the profit and loss statement was amended^81^. The change concerned three tax variables (current income tax, income tax adjusted on deferred tax assets and liabilities, and deferred tax assets and liabilities) that were consolidated in one variable. The change was effective for accounts filed starting from 2020, but the filers were allowed to use the new form in 2019 filings as well. The data does not allow us to distinguish between the firms using old and new forms in 2019 and we do not consolidate the tax lines in that year. Fifth, there is evidence that Russian firms, especially non-public ones, manipulate financial statements^28,73,82–84^, although later studies demonstrate the downward trend of falsification^85^. Sixth, the RFSD includes accounting-based measures of firm performance. However, certain research questions might require extraneous data that is unrelated to financial performance^86^.

## Supplementary information

Supplementary Materials

## Data Availability

The code used to build the RFSD is available at the dedicated GitHub repository (https://github.com/irlcode/RFSD). With access to the fee-based FNS API, it is possible to replicate our procedures to obtain, impute, and harmonize the financial statements data. To replicate the RFSD fully one would also need a panel of all active firms and their classification codes (can be obtained from FNS and Rosstat, respectively, as indicated in the Methods section), the pipeline for geo-coding is included in the repository. The repository also contains instructions for importing the RFSD data in R or Python environments, as well as three use cases. For those engaged in macroeconomic research, we present a replication of a study on the interest to cost of goods sold ratio of Russian firms by Mogilat *et al*.^39^, which was based on Interfax’s SPARK data. For scholars of industrial organization, we replicate the total factor productivity estimation of Kaukin and Zhemkova^31^, which employed Moody’s Ruslana data. For economic geographers, we offer a novel model-less house-level GDP spatialization that capitalizes on geocoding of firm addresses.
