# Supplementary material for: Russian Financial Statements Database: A firm-level collection of the universe of financial statements
Source: Sci Data. 2025 Jun 13;12:995. doi: 10.1038/s41597-025-05150-1 (PMC12166073; doi:10.1038/s41597-025-05150-1)
Supplement: Supplementary file 1 — Supplementary Materials [file 41597_2025_5150_MOESM1_ESM.pdf]

Supplementary Materials

Table A.1. Definition of variables in the RFSD

Variable	Suggested name	Description
		FIRM BASE INFO
year		Reporting period
inn		Taxpayer identifier (INN)
ogrn		Organization identifier (OGRN)
region		Region of incorporation
region_taxcode		Tax code of the region of incorporation
creation_date		Date of a firm's registration in EGRUL
dissolution_date		Date of a firm's exit from EGRUL
age		Firm's age in years in reporting period
		ELIGIBILITY
eligible		If a firm was eligible to file a financial statement in reporting period
exemption_criteria		Criteria of exemption from the obligation to file financial statement
financial		Firm is classified as a financial firm
		STATEMENT
filed		If a firm filed a statement for reporting period
imputed		If a statement was not filed but was (partially) reconstructed from the prior-years values reported in the next statement or the one after that
simplified		If a firm filed a statement using simplified (abbreviated) form
articulated		If values in a statement sum up to respective summarizing lines' values
totals_adjustment		If summarizing lines' values were missing or did not equate the sums of lines they summarized, and were therefore adjusted
outlier		Flag for statements with clearly anomalous revenue values
		CLASSIFICATION CODES
okved		A firm's industry code in terms of the Russian national classifier of economic activities (OKVED, NACE Rev.2-compatible)
okved_section		A firm's industry section in terms of the Russian national classifier of economic activities
okpo		A firm's type in terms of the Russian National Classifier of Enterprises and Organizations (OKPO)
okopf		A firm's legal form in terms of the Russian national classifier of organizational and legal forms (OKOPF)
okogu		An organization's type in terms of Russian national classifier of state authorities and administration (OKOGU)
okfc		A firm's ownership form in terms of the Russian national classifier of forms of ownership
oktmo		Code of municipal formation of a firm's incorporation in the Russian national classifier of municipal formations (OKTMO)
		LOCATION
lon		Longitude of a firm's address of incorporation
lat		Latitude of a firm's address of incorporation
geocoding_quality		Geocoding quality in terms of Nominatim Address Rank
		BALANCE SHEET
line_1100	B_noncurrent_assets	Total non-current assets

line_1110	B_intangible_assets	Intangible assets
line_1120	B_research_development	Research and development results
line_1130	B_intangible_exploration	Intangible exploration assets
line_1140	B_tangible_exploration	Tangible exploration assets
line_1150	B_fixed_assets	Fixed assets
line_1160	B_tangible_invest	Income investments in tangible assets
line_1170	B_fin_invest	Financial investments
line_1180	B_def_tax_assets	Deferred tax assets
line_1190	B_other_noncurrent_assets	Other non-current assets
line_1200	B_current_assets	Current assets
line_1210	B_inventories	Inventories
line_1220	B_vat_receivable	Value-added tax on acquired assets
line_1230	B_accounts_receivable	Accounts receivable
line_1240	B_fin_invest	Financial investments
line_1250	B_cash_equivalents	Cash and cash equivalents
line_1260	B_other_current	Other current assets
line_1300	B_total_equity	Total equity
line_1310	B_charter_capital	Charter capital (contributed capital, statutory fund, partners' contributions)
line_1320	B_treasury_shares	Treasury shares (repurchased from shareholders)
line_1340	B_reval_assets	Revaluation of non-current assets
line_1350	B_add_capital	Additional capital
line_1360	B_reserve_capital	Reserve capital
line_1370	B_retained_earnings	Retained earnings (uncovered loss)
line_1400	B_longterm_liab	Long-term liabilities
line_1410	B_longterm_debt	Long-term borrowings
line_1420	B_def_tax_liab	Deferred tax liabilities
line_1430	B_provision_liab	Provisions
line_1450	B_other_liab	Other liabilities
line_1500	B_shortterm_liab	Short-term liabilities
line_1510	B_shortterm_debt	Short-term borrowings
line_1520	B_shortterm_payables	Short-term payables
line_1530	B_def_income	Deferred income
line_1540	B_provision_liab	Provisions
line_1550	B_other_liab	Other liabilities
line_1600	B_assets	Assets
line_1700	B_liab	Liabilities

PROFIT AND LOSS STATEMENT

line_2110	PL_revenue	Revenue
line_2120	PL_cost_of_sales	Cost of sales
line_2100	PL_gross_profit	Gross profit (loss)
line_2210	PL_commercial_expenses	Commercial expenses
line_2220	PL_management_expenses	Management expenses
line_2200	PL_profit_from_sales	Profit (loss) from sales
line_2310	PL_income_participation	Income from participation in other organizations
line_2320	PL_interest_receivable	Interest receivable
line_2330	PL_interest_payable	Interest payable
line_2340	PL_other_income	Other income
line_2350	PL_other_expenses	Other expenses
line_2300	PL_before_tax	Profit (loss) before tax
line_2410	PL_income_tax	Income tax (Current income tax before 2019-2020)
line_2411	PL_current_income_tax	Current income tax
line_2412	PL_def_income_tax	Deferred income tax
line_2421	PL_tax_liab	Permanent tax liabilities (not used after 2019-2020)

line_2430	PL_change_def_tax_liab	Change in deferred tax liabilities (not used after 2019-2020)
line_2450	PL_change_def_tax_assets	Change in deferred tax assets (not used after 2019-2020)
line_2460	PL_other_factors	Other factors affecting the amount of net profit (fines, etc.)
line_2400	PL_net_profit	Net profit (loss)
line_2510	PL_reval	Result from revaluation of non-current assets which are not included in net profit (loss)
line_2520	PL_other_operations	Result from other operations which are not included in net profit (loss)
line_2530	PL_income_tax_operations	Income tax on operations which are not included in net profit (loss)
line_2500	PL_total	Total financial result for the period
line_2900	PL_basic_earnings_share	Basic earnings (loss) per share
line_2910	PL_diluted_earnings_share	Diluted earnings (loss) per share

Statement of changes in equity: Previous reporting period

line_3100	Epp_equity	The size of equity at the end of the year preceding the previous one
line_3210	Ep_incr	Total equity increase
line_3211	Ep_incr_net_profit	Equity increase due to net profit
line_3212	Ep_incr_asset_reval	Equity increase due to assets revaluation
line_3213	Ep_incr_income	Equity increase due to contributions from founders
line_3214	Ep_incr_add_share_issue	Equity increase due to additional shares issue
line_3215	Ep_incr_share_value	Equity increase due to increase in nominal value of shares
line_3216	Ep_incr_reorg	Equity increase due to reorganization
line_321x	Ep_incr_other	Other factors of equity increase
line_3220	Ep_decr	Total equity decrease
line_3221	Ep_decr_loss	Equity decrease due to loss
line_3222	Ep_decr_asset_reval	Equity decrease due to assets revaluation
line_3223	Ep_decr_expenses	Equity decrease due to expenses
line_3224	Ep_decr_share_value	Equity decrease due to decrease in nominal value of shares
line_3225	Ep_decr_shares_number	Equity decrease due to decrease in number of shares
line_3226	Ep_decr_reorg	Equity decrease due to legal entity reorganization
line_3227	Ep_decr_dividends	Equity decrease due to payment of dividends
line_322x	Ep_decr_special	Other factors of equity decrease listed in optional lines
line_3230	Ep_change_add	Change in additional equity
line_3240	Ep_change_reserve	Change in reserve equity
line_3200	Ep_equity	Equity amount as of December 31 of the previous year

Statement of changes in equity: Current reporting period

line_3310	E_incr	Total equity increase
line_3311	E_incr_net_profit	Equity increase due to net profit
line_3312	E_incr_asset_reval	Equity increase due to assets revaluation
line_3313	E_incr_income	Equity increase due to contributions from founders
line_3314	E_incr_add_share_issue	Equity increase due to additional shares issue
line_3315	E_incr_share_value	Equity increase due to increase in nominal value of shares
line_3316	E_incr_reorg	Equity increase due to reorganization
line_331x	E_incr_other	Other factors of equity increase
line_3320	E_decr	Total equity decrease
line_3321	E_decr_loss	Equity decrease due to loss
line_3322	E_decr_asset_reval	Equity decrease due to assets revaluation
line_3323	E_decr_expenses	Equity decrease due to expenses
line_3324	E_decr_share_value	Equity decrease due to decrease in nominal value of shares
line_3325	E_decr_shares_number	Equity decrease due to decrease in number of shares
line_3326	E_decr_reorg	Equity decrease due to legal entity reorganization
line_3327	E_decr_dividends	Equity decrease due to payment of dividends
line_332x	E_decr_special	Other factors of equity decrease listed in optional lines
line_3330	E_change_add	Change in additional equity
line_3340	E_change_reserve	Change in reserve equity

line_3300	E_equity	Equity amount as of December 31 of the reporting year
		**ADJUSTMENT DUE TO CHANGES IN ACCOUNTING POLICY AND CORRECTION OF ERRORS**
line_3400	ADJ_equity_before	Total equity Before Adjustments
line_3410	ADJ_policy	Adjustment Due to Change in Accounting Policy
line_3420	ADJ_error	Adjustment Due to Correction of Errors After Adjustment
line_3500	ADJ_equity_after	Total equity After Adjustments
line_3401	ADJ_undistr_profit_before	Amount of undistributed profit before adjustments
line_3411	ADJ_undistr_profit_policy	Adjustment of the amount of undistributed profit due to changes in accounting policy
line_3421	ADJ_undistr_profit_errors	Adjustment of the amount of undistributed profit due to correction of errors
line_3501	ADJ_undistr_profit_after	Amount of undistributed profit after adjustments
line_3402	ADJ_other_equity_before	Size of other equity items before adjustments
line_3412	ADJ_other_equity_policy	Adjustment of other equity items due to changes in accounting policy
line_3422	ADJ_other_equity_errors	Adjustment of other equity items due to correction of errors
line_3502	ADJ_other_equity_after	Size of other equity items after adjustments
		**NET ASSETS**
line_3600	NA_net_assets	Net assets
		**CASH FLOW STATEMENT: OPERATING ACTIVITIES**
line_4110	CFi_operating	Cash inflows from operating activities
line_4111	CFi_sales	From Sale of Products, Goods, Works, and Services
line_4112	CFi_payments	From Rental Payments, License Fees, Royalties, Commissions, and Other Similar Payments
line_4113	CFi_resale_invest	From Resale of Financial Investments
line_411x	CFi_firm_specific	Cash inflows stated in optional lines
line_4119	CFi_other	Other cash inflows
line_4120	CFo_operating	Cash outflows from operating activities
line_4121	CFo_materials	Payments to suppliers (contractors) for raw materials, goods, works, and services
line_4122	CFo_labor	Labor payments
line_4123	CFo_interest	Interest on debt obligations
line_4124	CFo_income_tax	Corporate income tax
line_412x	CFo_special	Cash outflows stated in optional lines
line_4129	CFo_other	Other payments
line_4100	CF_balance_operating	Balance of Cash Flows from Operating Activities
		**CASH FLOW STATEMENT: INVESTING ACTIVITIES**
line_4210	CFi_invest	Cash inflows from investments
line_4211	CFi_sale_noncurrent_assets	From Sale of Non-Current Assets (excluding Financial Investments)
line_4212	CFi_sale_shares	From Sale of Shares of Other Organizations (Equity Interests)
line_4213	CFi_loan_repayments	From Repayment of Loans Granted, From Sale of Debt Securities (Claims for Cash from Other Parties)
line_4214	CFi_dividends_interest	From Dividends, Interest on Debt Financial Investments, and Similar Inflows from Equity Participation in Other Organizations
line_421x	CFi_invest_special	Cash inflow from investment operations stated in optional lines
line_4219	CFi_invest_other	Other inflows from investments
line_4220	CFo_invest	Cash outflows from investments
line_4221	CFo_acquisition_assets	Cash outflows from acquisition, creation, modernization, reconstruction, and preparation for use of non-current assets
line_4222	CFo_acquisition_shares	Cash outflows from acquisition of shares of other organizations (equity interests)

line_4223	CFo_acquisition_debt	In connection with acquisition of debt securities (claims for cash from other parties), granting loans to other parties
line_4224	CFo_interest_payments	Interest on debt obligations included in the cost of investment assets
line_422x	CFo_invest_special	Cash outflows from investing listed in optional lines
line_4229	CFo_invest_other	Other payments because of investments
line_4200	CF_balance_invest	Balance of cash flows from investing activities

CASH FLOW STATEMENT: FINANCIAL OPERATIONS

line_4310	CFi_fin	Cash inflows from financial operations
line_4311	CFi_loans	Receipt of loans and borrowings
line_4312	CFi_owner_contributions	Cash contributions from owners (participants)
line_4313	CFi_share_issuance	From issuance of shares, increase in ownership interests
line_4314	CFi_bond_issuance	From issuance of bonds, promissory notes, and other debt securities
line_431x	CFi_fin_special	Cash inflows from financial operations stated in optional lines
line_4319	CFi_fin_other	Other inflows from financial operations
line_4320	CFo_fin	Cash outflows from financial operations
line_4321	CFo_payments_owners	To owners (participants) in connection with buyback of shares (ownership interests) or their exit from the organization
line_4322	CFo_payments_dividends	For payment of dividends and other profit distribution payments to owners (participants)
line_4323	CFo_debt_repayments	In connection with redemption (buyback) of promissory notes and other debt securities, repayment of loans and borrowings
line_432x	CFo_fin_special	Cash outflows from financial activities stated in optional lines
line_4329	CFo_fin_other	Other cash outflows from financial activities
line_4300	CF_balance_fin	Balance of cash flows from financing activities
line_4400	CF_balance	Balance of cash flows for the reporting period
line_4450	C_balance_start	Balance of cash and cash equivalents at the start of the reporting period
line_4500	C_balance_end	Ending balance of cash and cash equivalents at the end of the reporting period
line_4490	C_foreign_currency_impact	Impact of foreign currency exchange rate changes relative to the ruble

STATEMENT ON THE PROPER USE OF FUNDS RECEIVED

line_6100	PU_start	Beginning balance of funds at the start of the reporting year
line_6210	PU_entrance	Entrance fees
line_6215	PU_membership_fees	Membership fees
line_6220	PU_designated	Designated contributions
line_6230	PU_voluntary	Voluntary property contributions and donations
line_6240	PU_income_activities	Profit from income-generating activities of the organization
line_6250	PU_income_other	Other
line_6200	PU_total_received	Total funds received
line_6310	PU_designated	Expenses for designated activities
line_6311	PU_aid	Social and charitable assistance
line_6312	PU_conference	Expenses for conducting conferences, meetings, seminars
line_6313	PU_other_events	Other activities
line_6320	PU_administrative	Administrative expenses
line_6321	PU_labor	Labor-related expenses (including accruals)
line_6322	PU_nonlabor	Payments not related to labor
line_6323	PU_travel	Expenses for business trips and travel
line_6324	PU_maintenance	Maintenance of premises, buildings, vehicles, and other property (excluding repairs)
line_6325	PU_repairs	Repairs of fixed assets and other property
line_6326	PU_other_administrative	Other administrative expenses
line_6330	PU_acquisition_assets	Acquisition of fixed assets, inventory, and other property
line_6350	PU_other_expenses	Other
line_6300	PU_total_expenses	Total funds used

line_6400 PU_remaining	Funds at the end of the reporting period

Table A.2. Official articulation equations used in this paper

Summarizing line number		Articulation equation
		FULL STATEMENTS
Balance sheet		
1100	=	1110 + 1120 + 1130 + 1140 + 1150 + 1160 + 1170 + 1180 + 1190
1200	=	1210 + 1220 + 1230 + 1240 + 1250 + 1260
1300	=	1310 + 1320 + 1330 + 1340 + 1350 + 1360 + 1370
1400	=	1410 + 1420 + 1430 + 1450
1500	=	1510 + 1520 + 1530 + 1540 + 1550
1600	=	1200 + 1100
1600	=	1700
1700	=	1300 + 1400 + 1500
Profit and loss statement		
2100	=	2110 − 2120
2200	=	2100 − 2210 − 2220
2300	=	2200 − 2310 + 2320 − 2330 + 2340 − 2350
Cash flow statement		
4100	=	4110 − 4120
4110	=	4111 + 4112 + 4113 + 4114 + 4116 + 4119 + optional decoding lines
4120	=	4121 + 4122 + 4123 + 4124 + 4126 + 4129 + optional decoding lines
4200	=	4210 − 4220
4210	=	4211 + 4212 + 4213 + 4214 + 4216 + 4219 + optional decoding lines
4220	=	4221 + 4222 + 4223 + 4224 + 4226 + 4229 + optional decoding lines
4300	=	4310 − 4320
4310	=	4311 + 4312 + 4313 + 4314 + 4316 + 4319 + optional decoding lines
4320	=	4321 + 4322 + 4323 + 4324 + 4326 + 4329 + optional decoding lines
4400	=	4100 + 4200 + 4300
4500	=	4400 + 4450 + 4490
		SIMPLIFIED STATEMENTS
Balance sheet		
1600	=	1150 + 1170 + 1210 + 1250 + 1230
1600	=	1700
1700	=	1300 + 1410 + 1450 + 1510 + 1520 + 1550
Profit and loss statement		
2400	=	2110 − 2120 − 2330 + 2340 − 2350 − 2410
